# Green synthesis and synergistic catalytic effect ofAg/reduced graphene oxide nanocomposite

**DOI:** 10.1186/1556-276X-9-484

**Published:** 2014-09-11

**Authors:** Kai-Chih Hsu, Dong-Hwang Chen

**Affiliations:** 1 Department of Chemical Engineering, National Cheng Kung University, Tainan 701, Taiwan

**Keywords:** Silver nanoparticles, Reduced graphene oxide, Catalyst, Green synthesis, Synergistic effect

## Abstract

A nanocomposite of silver nanoparticles and reduced graphene oxide (Ag/rGO) has been developed as a catalyst for the reduction of 4-nitrophenol (4-NP) to 4-aminophenol (4-AP) with sodium borohydride, owing to the larger specific surface area and synergistic effect of rGO. A facile and rapid microwave-assisted green route has been used for the uniform deposition of Ag nanoparticles and the reduction of graphene oxide simultaneously with l-arginine as the reducing agent. The resulting Ag/rGO nanocomposite contained about 51 wt% of Ag, and the Ag nanoparticles deposited on the surface of rGO had a mean diameter of 8.6 ± 3.5 nm. Also, the Ag/rGO nanocomposite exhibited excellent catalytic activity and stability toward the reduction of 4-NP to 4-AP with sodium borohydride. The reduction reaction obeyed the pseudo-first-order kinetics. The rate constants increased not only with the increase of temperature and catalyst amount but also with the increase of initial 4-NP concentration, revealing that the support rGO could enhance the catalytic activity via a synergistic effect. A mechanism for the catalytic reduction of 4-NP with NaBH_4_ by Ag/rGO nanocomposite via both the liquid-phase and solid-phase routes has been suggested.

## Background

The removal of 4-nitrophenol (4-NP) has received continuous attention in the past decades because it is a common organic pollutant in industrial wastewater. Many processes such as adsorption, microbial degradation, photocatalytic degradation, Fenton method, and electrochemical treatment have been developed [1]. Furthermore, 4-NP can be utilized as a precursor of 4-aminophenol (4-AP). 4-AP is not only an intermediate in the syntheses of many analgesic/antipyretic drugs but also can be regarded as a corrosion inhibitor, photographic developer, hair-dyeing agent, and anticorrosion lubricant [2-4]. So, a great deal of efforts have been made on the reduction of 4-NP to 4-AP. Among them, the borohydride reduction of 4-NP to 4-AP by metal nanoparticles such as Au, Ag, Pt, Pd, and Pt-Ni is particularly attractive because this reaction can be performed in aqueous solution under mild condition [5-10]. However, for the sake of energy saving, safe operation, and avoiding the use of organic solvents, the development of more appropriate processes for the reduction of 4-NP to 4-AP in aqueous solutions under mild condition is still in demand.

On the other hand, graphene which is a two-dimensional single-layer carbon sheet has attracted great interest recently in various fields of science and engineering because of its unique electrical, optical, thermal, and mechanical properties [11-15]. Although a lot of methods have been developed for the preparation of graphene sheets, the most suitable and efficient approach was the solution-based chemical reduction of exfoliated graphite oxide to reduced graphene oxide (rGO) because of its low cost and facile synthetic nature in a controlled, scalable, and reproducible manner [16,17]. Graphite oxide can be readily dispersed in water to yield the stable dispersions of graphene oxide (GO) by simple sonication, owing to the presence of oxygen-containing functional groups such as hydroxyl, epoxide, and carboxyl moieties [18]. Furthermore, these oxygen-containing functional groups can act as nucleation centers or anchoring sites for the attachment of nanoparticles [19]. They limited the growth of nanoparticles and improved the stability and dispersion of nanoparticles on GO or rGO. The attached nanoparticles are also helpful for enlarging the interplanar spacing of the GO or rGO in solid state, maintaining the excellent properties of individual GO or rGO sheets, and avoiding the aggregation of GO or rGO sheets into graphitic structure [20,21]. Because of its large specific surface area and the above advantages, GO and rGO have been widely used as the supports for the attachment of nanoparticles to yield the nanocomposites for various applications.

According to the above, in this work, we attempted to fabricate the nanocomposite of Ag nanoparticles and rGO (Ag/rGO) for the catalytic reduction of 4-NP to 4-AP. Ag nanoparticles were chosen because they were cheaper as compared to other noble metal catalysts such as Au, Pt, and Pd [5-10,22,23]. Such a nanocomposite not only could be used in the catalytic field but also has been widely applied in antibacterial agent, electrochemical analysis, surface-enhanced Raman scattering (SERS), and so on [24-31]. Recently, we have developed a facile and rapid microwave-assisted green route to fabricate the Ag/rGO nanocomposite as a SERS substrate with high uniformity by using l-arginine as the reducing agent [32]. The average size and content of Ag nanoparticles could be easily controlled by adjusting the cycle number of microwave irradiation. In that work, the formation of larger Ag nanoparticles which favored the enhancement of SERS intensity was desired. However, for the catalytic application, it is known that smaller nanoparticles usually exhibit higher specific catalytic activity. So, in this study, by modifying the condition of the l-arginine-based microwave-assisted green route, smaller Ag nanoparticles were uniformly deposited on rGO to fabricate the Ag/rGO nanocomposite for the catalytic reduction of 4-NP to 4-AP with sodium borohydride. As compared to other studies on the synthesis of Ag/rGO nanocomposites for catalytic reduction of 4-NP, the resulting Ag/rGO nanocomposite in this work had significantly higher content of Ag nanoparticles on rGO [24-26]. In addition, from the effect of 4-NP concentration, the synergistic effect of rGO was also demonstrated.

## Methods

Graphite powder (99.9 %) was obtained from Bay Carbon (Bay City, MI, USA). Potassium manganite (VII) and sodium nitrate were purchased from J. T. Baker (Center Valley, PA, USA). Sulfuric acid was supplied by Panreac (Barcelona, Spain). Hydrogen peroxide was a product of Showa (Tokyo, Japan). Sulfuric acid was obtained from Merck (Whitehouse Station, NJ, USA). l-Arginine was supplied by Sigma-Aldrich (St. Louis, MO, USA). Silver nitrate and 4-nitrophenol were obtained from Alfa Aesar (Ward Hill, MA, USA). Sodium borohydride was the product of Aldrich. All chemicals were of guaranteed or analytical grade reagents commercially available and used without further purification. The water used throughout this work was the reagent grade water produced by a Milli-Q SP ultra-pure-water purification system of Nihon Millipore Ltd., Tokyo, Japan.

GO was prepared from purified natural graphite by a modified Hummers method [20]. The graphite powder (1.5 g) and NaNO_3_ (0.75 g) were added to the concentrated H_2_SO_4_ (18 M, 37 mL) in an ice bath. KMnO_4_ (4.5 g) was added gradually under stirring. The mixture was then stirred at 35°C for 24 h. Then, deionized water (70 mL) was slowly added to the mixture, followed by stirring of the mixture at 98°C for 15 min. The suspension was further diluted to 110 mL and stirred for another 30 min. The reaction was terminated by adding H_2_O_2_ (3.7 mL, 35 wt%) under stirring at room temperature, followed by washing with deionized water several times.

Ag/rGO nanocomposite was synthesized by a facile, rapid and green process. Firstly, 15 mg of graphite oxide was dispersed in 20 mL of deionized water by ultrasonication to form a stable GO colloid solution, and then mixed with 10 mL of AgNO_3_ (180 mM) and l-arginine (60 mg/mL) solution. Next, the solution was transferred into a Teflon beaker and then reduced by three cycles of microwave irradiation (2.45 GHz, 900 W). Each cycle included 50 s ON and 10 s OFF. The product (denoted as 3C) was collected by centrifugation, then washed several times with deionized water, and finally dried in a vacuum oven at 35°C. Following the above procedures in the absence of AgNO_3_, rGO was prepared to confirm the reduction of GO by l-arginine and for comparison with Ag/rGO. Moreover, to investigate the effect of size and content of Ag nanoparticles on rGO, another two products 1C and 5C were also obtained under the same condition by one and five cycles of microwave irradiation, respectively.

The particle size and composition were determined by transmission electron microscopy (TEM) and energy dispersive X-ray (EDX) spectroscopy on a high-resolution field emission transmission electron microscopy (HRTEM, JEOL Model JEM-2100 F, JEOL Ltd., Tokyo, Japan). The high-resolution TEM (HRTEM) image and selected area electron diffraction (SAED) pattern were obtained by a JEOL Model JEM-2100 F electron microscope at 200 kV. The Ag content of Ag/rGO nanocomposite was also determined by dissolving the sample in a concentrated HCl solution and analyzing the solution composition using a GBC Model SDS-270 atomic absorption spectrometer (AAS; GBC Scientific, Braeside, Australia). The UV–vis absorption spectra of the resultant colloid solutions were monitored by a JASCO model V-570 UV/VIS/NIR spectrophotometer (JASCO, Tokyo, Japan). The crystalline structures were characterized by X-ray diffraction (XRD) analysis on a Shimadzu model RX-III X-ray diffractometer (Shimadzu Corporation, Kyoto, Japan) at 40 kV and 30 mA with CuKα radiation (*λ* = 0.1542 nm). Raman scattering was performed on a Thermo Fisher Scientific DXR Raman microscope using a 532-nm laser source (Thermo Fisher Scientific, Waltham, MA, USA). The X-ray photoelectron spectroscopy (XPS) measurements were performed on a Kratos AXIS Ultra DLD photoelectron spectrophotometer (Kratos Analytical Ltd, Manchester, UK) with an achromatic Mg/Al X-ray source at 450 W. Unless otherwise specified, the above characterization was done for the Ag/rGO nanocomposite 3C.

For the catalytic reduction of 4-nitrophenol, in general, an appropriate amount of Ag/rGO nanocomposite was added to the aqueous solution containing 4-NP and NaBH_4_ at the desired temperature to start the reaction. The bright yellow color of the solution gradually vanished, indicating the reduction of 4-NP. The variation of 4-NP concentration with time was monitored spectrophotometrically at a wavelength of 400 nm. The initial concentration ratio of NaBH_4_ to 4-NP was fixed at 100 so that the concentration of NaBH_4_ could be considered as a constant during the reaction. For the reusability study, the Ag/rGO nanocomposite was collected by centrifugation, washed with deionized water, and then reused. Unless otherwise specified, the Ag/rGO nanocomposite used for the catalytic reduction of 4-NP was the product 3C.

## Results and discussion

**Figure 1:**
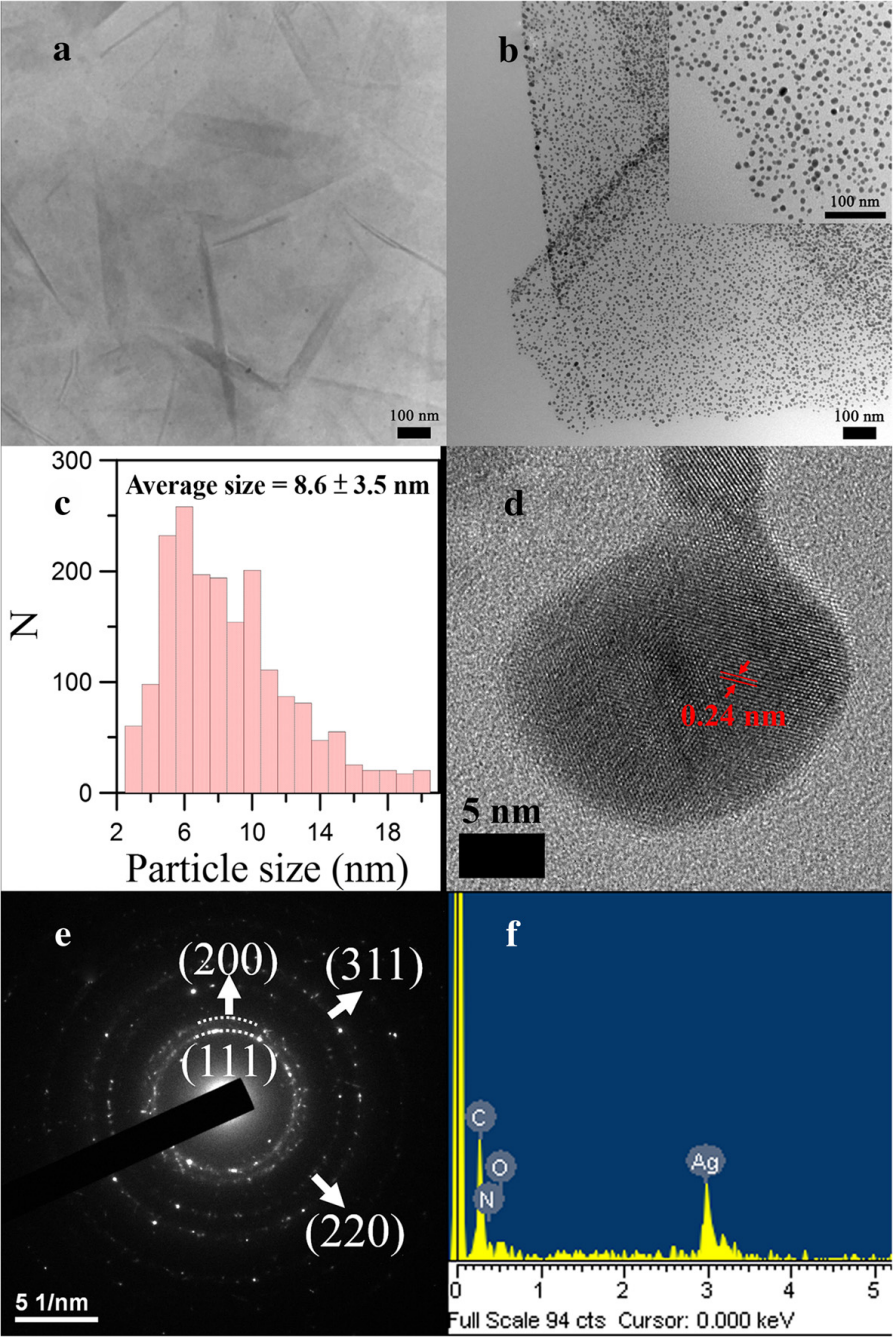
**Ag/rGO nanocomposites.** Typical TEM images of the Ag/rGO nanocomposites obtained in the **(a)** absence and **(b)** presence of l-arginine under microwave irradiation. **(c)** Particle size distribution of Ag nanoparticles on rGO. **(d)** HRTEM image, **(e)** SAED pattern, and **(f)** EDX spectrum of Ag/rGO nanocomposite. The inset in **(b)** is a magnified image.

**Figure 2:**
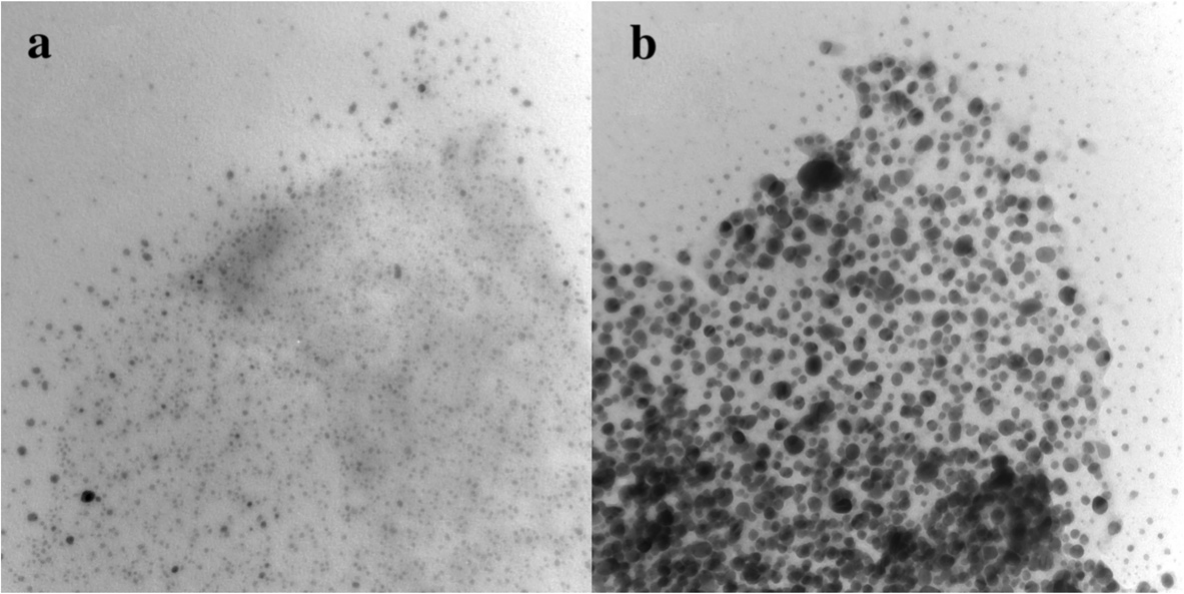
Typical TEM images of Ag/rGO nanocomposites (a) 1C and (b) 5C.

Figure 1a, b shows the typical TEM images of the Ag/rGO nanocomposites obtained in the absence and presence of l-arginine under microwave irradiation, respectively. It was found that Ag nanoparticles were not significantly formed on rGO in the absence of l-arginine. However, in the presence of l-arginine, Ag nanoparticles were uniformly deposited on rGO. This revealed the necessity of l-arginine as a reducing agent for the formation of Ag nanoparticles. From the particle size distribution as indicated in Figure 1c, the mean diameter of Ag nanoparticles on rGO could be estimated to be 8.6 ± 3.5 nm. In addition, Figure 1d shows the HRTEM image. An interlayer spacing of 0.24 nm which related to the (111) plane of face-centered cubic (fcc) Ag could be observed. Also, the SAED pattern as indicated in Figure 1e showed the characteristic rings for the (111), (200), (220), and (311) planes of fcc Ag. Both the results revealed that the Ag nanoparticles decorated on rGO had a fcc structure. Moreover, by EDX analysis as indicated in Figure 1f, the presence of Ag and N elements confirmed the deposition of Ag nanoparticles and the capping of l-arginine. Also, the weight percentage of Ag in the product was determined to be 51.2 wt%. To confirm the composition, the Ag content was also determined to be 50.7 wt% by AAS. This result was in good agreement with that by EDX analysis and revealed that the Ag content of Ag/rGO nanocomposite was about 51 wt%. As compared to the early studies on the synthesis of Ag/rGO nanocomposites for catalytic reduction of 4-NP, the Ag/rGO nanocomposite obtained in this work had significantly higher Ag content [24-27].Figure 2 shows the typical TEM images of Ag/rGO nanocomposites 1C and 5C. It was found that the products 1C and 5C had mean diameters of 3.12 ± 2.15 nm and 11.7 ± 8.15 nm, respectively. Also, by EDX analysis, their Ag contents were determined to be 30.6 and 85.9 wt%, respectively. This revealed that the size and content of Ag nanoparticles for the Ag/rGO nanocomposite increased with the increase in the cycle number of microwave irradiation. Because the product 3C had the highest catalytic activity for the reduction of 4-NP (to be shown later), the following characterization and catalytic reaction were conducted for the product 3C unless otherwise specified.

**Figure 3:**
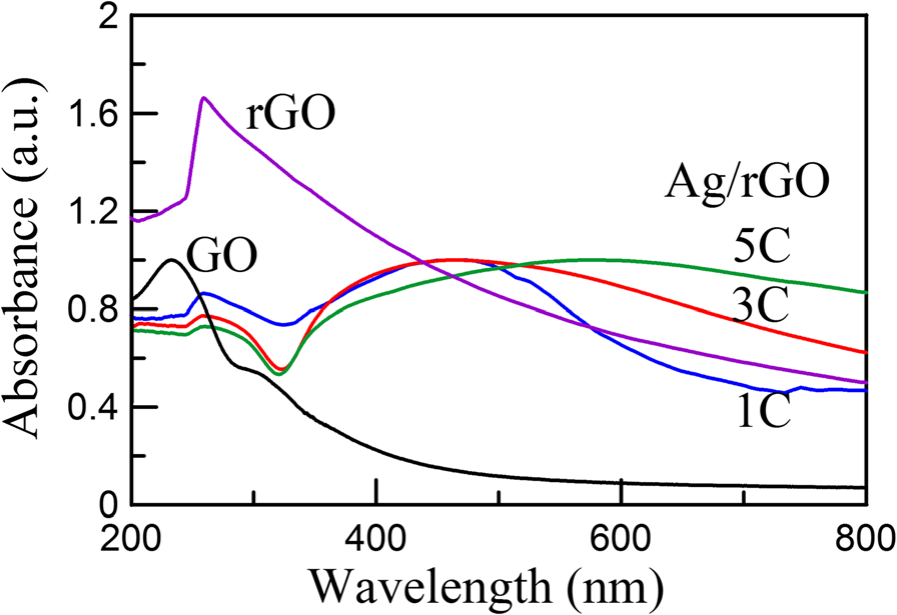
UV–vis spectra of GO, rGO, and Ag/rGO nanocomposites 1C, 3C and 5C.

Figure 3 shows the UV–vis absorption spectrum of GO, rGO, and Ag/rGO nanocomposites 1C, 3C, and 5C. Two characteristic peaks of GO could be observed. The peak at 233 nm and the shoulder peak around 300 nm related to the absorption of C-C bond and C = O bond, respectively [26,27,33-35]. The characteristic peak of rGO was observed at about 260 nm. Because it was slightly lower than 268 nm, it was suggested that GO was partially reduced to rGO [27]. The absorption spectra of Ag/rGO nanocomposites 1C, 3C, and 5C not only showed the characteristic peak of rGO at about 260 nm but also exhibited broad characteristic peaks of Ag nanoparticles, confirming the successful deposition of Ag nanoparticles. Furthermore, the characteristic peak of Ag nanoparticles on rGO became broader and red-shifted more significantly with the increase in the cycle number of microwave irradiation. This might be due to the substrate effect and the increase in the surface coverage of rGO by Ag nanoparticles [36,37].

**Figure 4:**
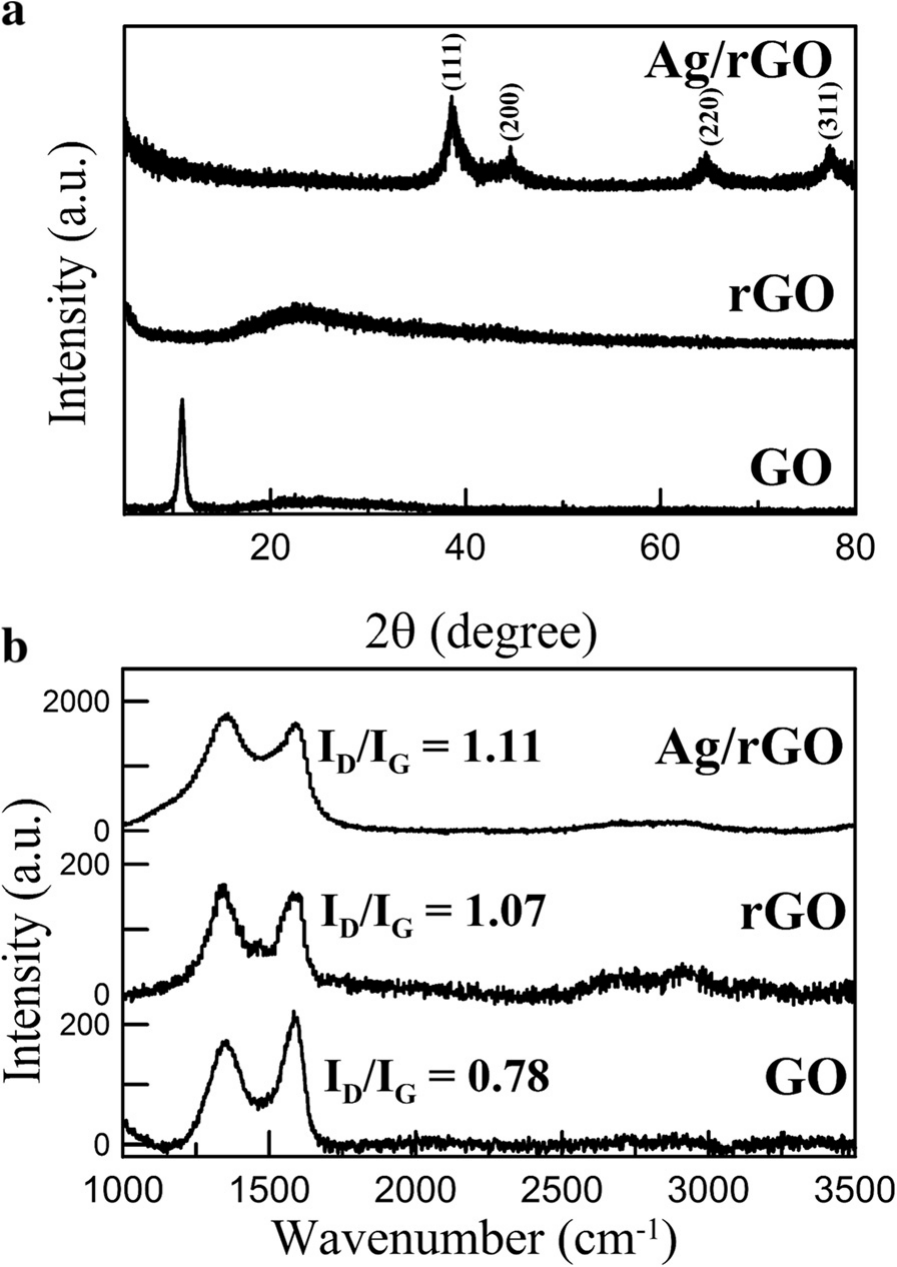
XRD patterns (a) and Raman spectra (b) of GO, rGO, and Ag/rGO nanocomposite.

Figure 4a shows the XRD patterns of GO, rGO, and Ag/rGO nanocomposite. The characteristic peaks of GO and rGO were observed at 2*θ* = 10.56° and 24.18°, respectively, confirming GO has been reduced to rGO successfully by l-arginine under microwave irradiation. For the Ag/rGO nanocomposite, the characteristic peaks at 2*θ* = 38.02°, 44.13°, 64.44°, and 77.31° related to the (111), (200), (220), and (311) planes of fcc Ag, respectively. They confirmed the formation of Ag nanoparticles on rGO. Furthermore, according to the Scherrer equation, the grain size of Ag nanoparticles on rGO could be estimated to be 5.6 nm. This was consistent with the mean diameter of Ag nanoparticles on rGO (i.e., 8.6 ± 3.5 nm) as indicated in Figure 1c.

The Raman spectra of GO, rGO, and Ag/rGO nanocomposite are shown in Figure 4b. Two prominent peaks corresponding to the G and D bands of graphene were observed clearly. The G band is usually assigned to the E_2g_ phonon of C sp^2^ atoms, while the D band originates from a breathing *κ*-point phonon with A_1g_ symmetry and relates to local defects and disorders [38-40]. The intensity ratio of D band to G band (*I*_D_/*I*_G_) is correlative with the average size of sp^2^ domains [41]. Higher *I*_D_/*I*_G_ ratio means the smaller size of sp^2^ domains. From Figure 4b, the *I*_D_/*I*_G_ ratios for GO, rGO, and Ag/rGO were 0.78, 1.07, and 1.11, respectively. It was reasonable that the *I*_D_/*I*_G_ ratios of rGO and Ag/rGO nanocomposite were larger than that of GO because the conjugated graphene network (sp^2^ carbon) would be re-established after chemical reduction of GO but the size of the re-established graphene network was usually smaller than the original graphite layer, leading to the increase of *I*_D_/*I*_G_ ratio [42]. Thus, it could be concluded that silver ions and GO were reduced simultaneously by l-arginine. In addition, it was noted that the intensities of the D band and G band for Ag/rGO were larger than those for rGO. This was due to the Ag nanoparticles deposited on rGO which enhanced the Raman signal via SERS effect [31,32]. Also, the *I*_D_/*I*_G_ ratio of Ag/rGO was slightly larger than that of rGO. This might be due to the increase in the number of defects after the deposition of Ag nanoparticles on rGO.

**Figure 5:**
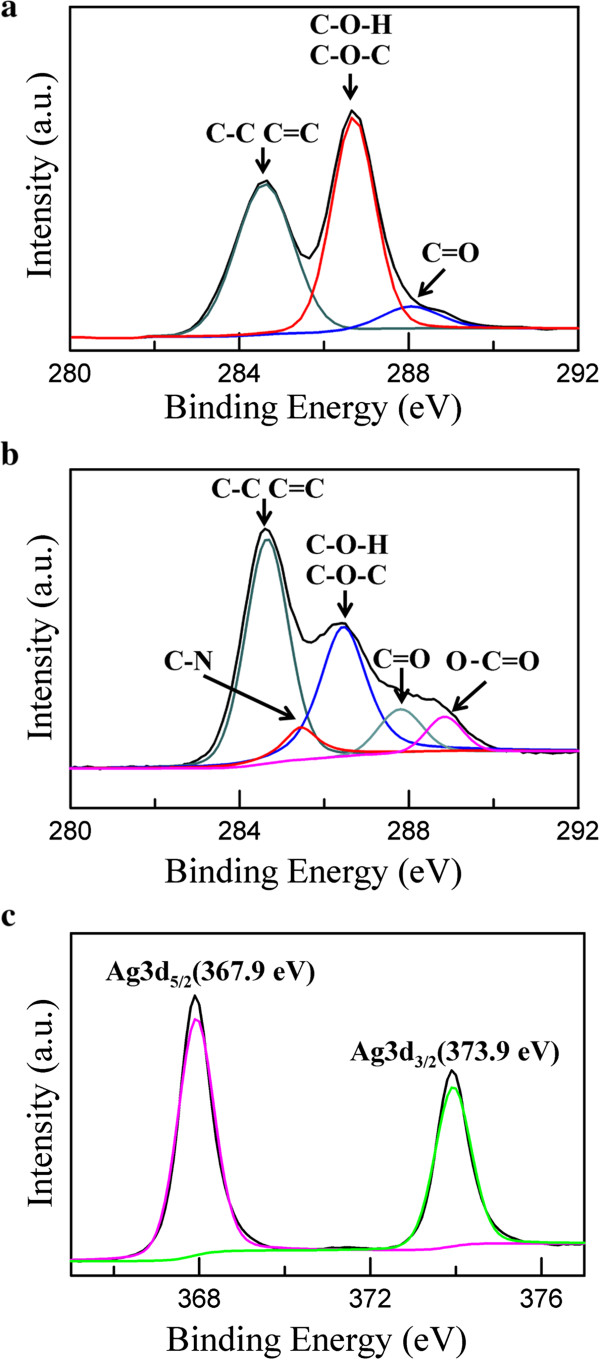
XPS spectra of (a) C1s of GO and (b) C1s and (c) Ag3d of Ag/rGO nanocomposite.

Figure 5 shows the XPS spectra of GO and Ag/rGO nanocomposite. In Figure 5a, the C1s XPS spectrum of GO at 280 to 292 eV showed the characteristic peaks of C-C, C = C, C-O-H, C-O-C, and C = O. They could be attributed to the presence of epoxide, hydroxyl, and carboxyl groups [41,42]. In Figure 5b, the peak intensity of C1s which related to oxygenated functional groups (C-O-H and C-O-C) showed a significant decrease, confirming that most of the epoxide, hydroxyl, and carboxyl functional groups were removed and GO has been successfully reduced [41,42]. It was noted that two new characteristic peaks of C-N and O-C = O were observed. They might be due to the l-arginine capped on the surface of Ag/rGO. Figure 5c shows the XPS signature of the Ag 3d doublet (3d_5/2_ and 3d_3/2_) for the Ag nanoparticles deposited on rGO. The Ag 3d_5/2_ and 3d_3/2_ peaks appeared at 367.9 and 373.9 eV, respectively. They shifted to the lower binding energy as compared to the characteristic peaks for silver metal at 368.2 and 374.2 eV. Similar phenomenon was also observed in the study of Li and Liu [33]. They attributed the negative shift to the electron transfer from metallic Ag to the graphene sheets owing to the smaller work function of Ag (4.2 eV) than graphene (4.48 eV) and the interaction between the Ag and the carboxyl (C = O) groups on the edge of graphene sheets because the binding energy of the higher ionic state of Ag was lower than that of zero-valent Ag [33]. In addition, the Ag 3d_5/2_ binding energies of Ag, Ag_2_O, and AgO were 368.2, 367.4, and 367.8 eV, respectively [43]. The slight oxidation on the surface of Ag nanoparticles might be the other reason for the negative shift of Ag 3d_3/2_ and Ag 3d_5/2_ binding energy.

**Figure 6:**
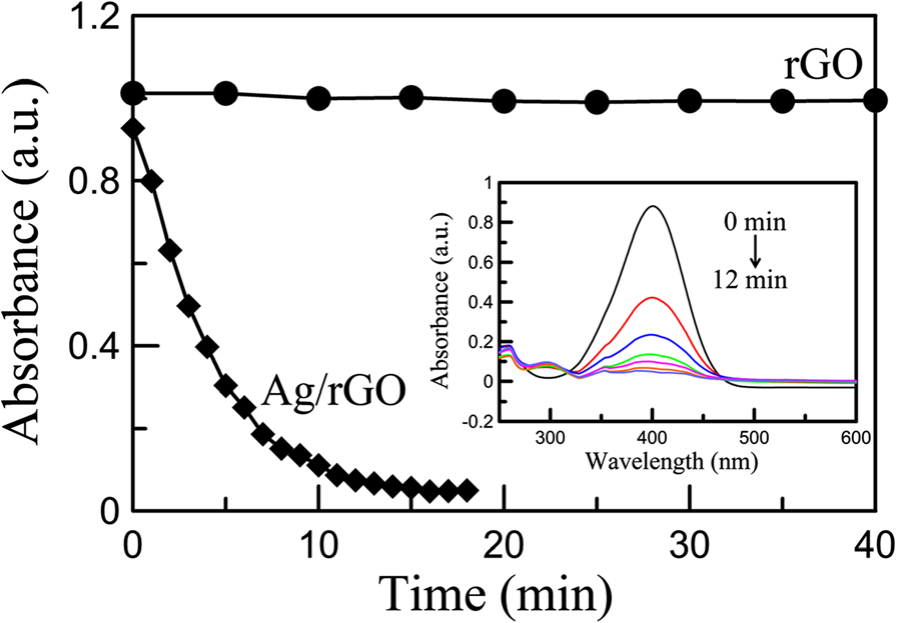
**Absorbance vs. time for reduction of 4-NP with NaBH4.** Time dependence of the absorbance at 400 nm for the catalytic reduction of 4-NP with NaBH_4_ by rGO or Ag/rGO nanocomposite. The inset indicates the variation of UV–vis absorption spectra with time for the catalytic reduction of 4-NP with NaBH_4_ by Ag/rGO nanocomposite.

To demonstrate the catalytic ability, the reduction of 4-NP (0.05 mM) with NaBH_4_ at 25°C in the presence of rGO or Ag/rGO nanocomposite (0.25 mg/100 mL) was examined. Figure 6 shows that the absorbance at 400 nm decreased to about zero rapidly within 12 min in the presence of Ag/rGO nanocomposite but had no significant variation in the presence of rGO. This revealed that rGO had no significant catalytic ability for the reduction of 4-NP, whereas the Ag/rGO nanocomposite possessed good catalytic ability for the reduction of 4-NP and the catalytic ability was due to the deposited Ag nanoparticles.

**Figure 7:**
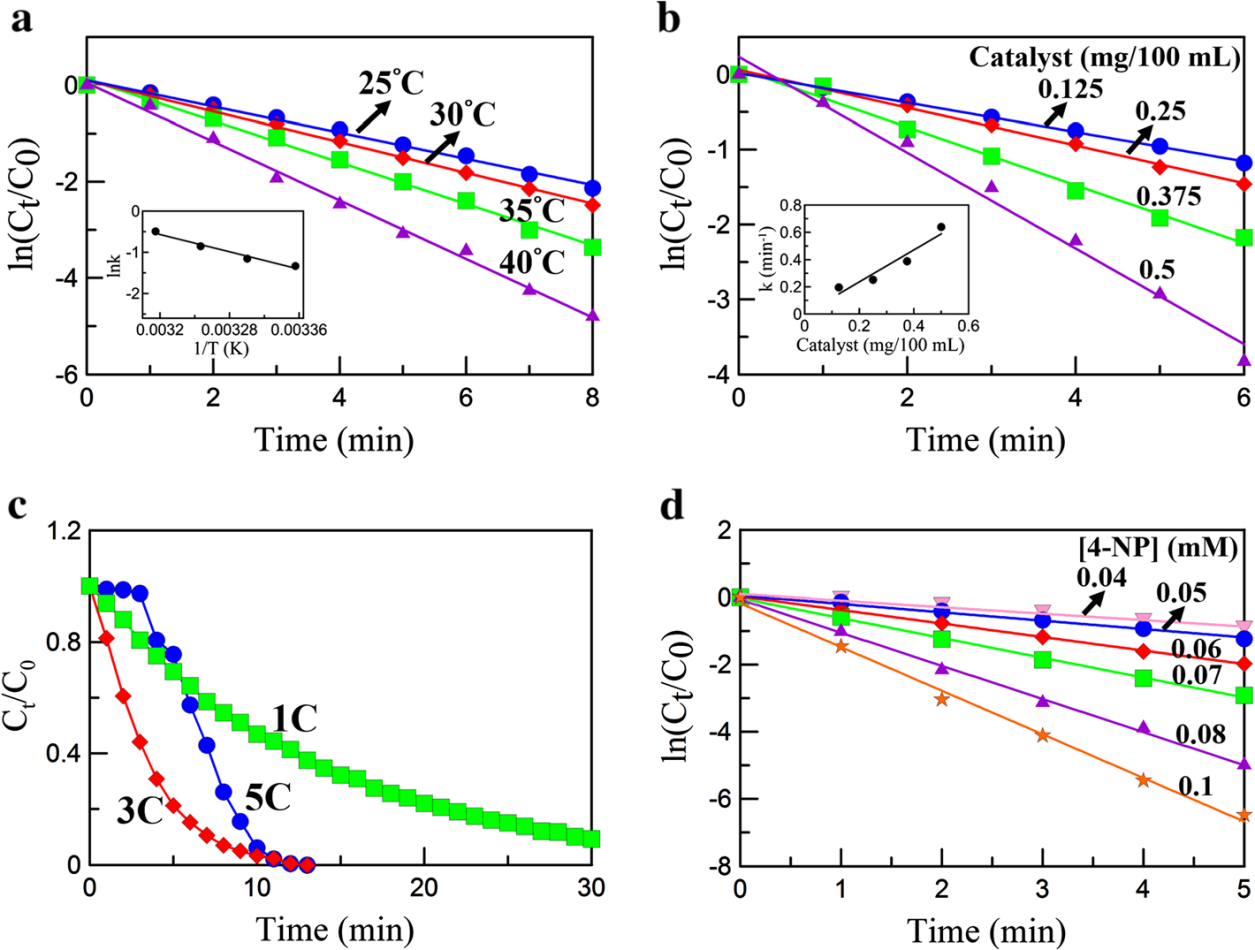
**ln (*C***_***t***_**/*C***_**0**_**) versus time.** Plots of ln (*C*_*t*_/*C*_0_) versus time for the catalytic reduction of 4-NP with NaBH_4_**(a)** by Ag/rGO nanocomposite at different temperatures; **(b)** by different amounts of Ag/rGO nanocomposite; **(c)** by Ag/rGO nanocomposites 1C, 3C, and 5C; and **(d)** by Ag/rGO nanocomposite at different initial 4-NP concentrations. *C*_0_ and *C*_*t*_ denote the concentrations of 4-NP when times were 0 and *t*, respectively. [NaBH_4_]/[4-NP] = 100. Unless otherwise specified in the figures, [4-NP] = 0.05 mM, catalyst amount = 0.25 mg/100 mL, temperature = 25°C, and Ag/rGO nanocomposite = 3C. The inset in **(a)** indicates the corresponding Arrhenius plot. The inset in **(b)** indicates the relationship between the pseudo-first-order rate constant and catalyst amount.

The effect of temperature on the catalytic reduction of 4-NP (0.05 mM) with NaBH_4_ by Ag/rGO nanocomposite (0.25 mg/100 mL) is shown in Figure 7a, in which *C*_0_ and *C*_*t*_ denote the initial 4-NP concentration and the 4-NP concentration at the time *t*, respectively. It was found that the reaction followed the pseudo-first-order kinetics. The corresponding pseudo-first-order rate constants (*k*), *R*^2^, and turnover frequencies (TOF) are listed in Table 1. Obviously, the reduction rate increased with the increase of temperature. By the Arrhenius plot as indicated in the inset of Figure 7a, the activation energy could be calculated to be 43.7 kJ/mol.

**Table 1:** **Pseudo-first-order rate constants and TOF for reduction of 4-NP with NaBH**_**4 **_**in the presence of Ag/rGO nanocomposite**

***W***_**cat**_	**[4-NP]**	**Temperature**	***k***	***R***^**2**^	**TOF (mmol/g/s)**
**(mg/100 mL)**	**(mM)**	**(°C)**	**(min**^**-1**^**)**		
0.25	0.05	40	0.609	0.9590	0.203
0.25	0.05	35	0.431	0.9956	0.144
0.25	0.05	30	0.319	0.9972	0.106
0.25	0.05	25	0.271	0.9936	0.090
0.125	0.05	25	0.196	0.9991	0.131
0.375	0.05	25	0.388	0.9896	0.086
0.50	0.05	25	0.639	0.9856	0.106
0.25	0.04	25	0.192	0.9695	0.051
0.25	0.06	25	0.403	0.9991	0.161
0.25	0.07	25	0.591	0.9983	0.276
0.25	0.08	25	0.988	0.9972	0.527
0.25	0.10	25	1.301	0.9951	0.867

Figure 7b indicates the catalytic reduction of 4-NP (0.05 mM) with NaBH_4_ at 25°C by different amounts of Ag/rGO nanocomposite (0.125 to 0.5 mg/100 mL). It was obvious that the reduction rate increased with the increase of the catalyst amount. The corresponding pseudo-first-order rate constants, *R*^2^, and turnover frequencies are also listed in Table 1. Because the pseudo-first-order rate constant increased linearly with the catalyst amount as indicated in the inset of Figure 7b, the role of Ag/rGO nanocomposite as the catalyst for the reduction of 4-NP with NaBH_4_ could be confirmed.

It was noted that the size and content of Ag nanoparticles on rGO should also influence the catalytic ability of Ag/rGO nanocomposite. Figure 7c shows the catalytic reduction of 4-NP (0.05 mM) with NaBH_4_ at 25°C by Ag/rGO nanocomposites 1C, 3C, and 5C (0.25 mg/100 mL). It was found that the catalytic activities of Ag/rGO nanocomposites increased in the following sequence: 1C < 5C < 3C. This revealed that the catalytic ability indeed depended on both the size and content of Ag nanoparticles on rGO. It is known that the specific activities of metal catalysts usually decrease with the increase of their size. Although increasing Ag content might increase the catalytic activity, too high Ag content might lead to the decrease of catalytic activity owing to the larger particle size and slight particle aggregation. So, in this work, the Ag/rGO nanocomposite 3C was found to be a better product for the catalytic reduction of 4-NP.

Figure 7d shows the effect of initial 4-NP concentration on the catalytic reduction of 4-NP (0.04 to 0.1 mM) with NaBH_4_ by Ag/rGO nanocomposite (0.25 mg/100 mL) at 25°C. The corresponding pseudo-first-order rate constants, *R*^2^, and turnover frequencies are listed in Table 1. It was found that the reaction rate increased with the increase of the initial 4-NP concentration. This phenomenon was different from some earlier studies and suggested that the use of rGO as the catalyst support might be helpful for the increase of catalytic activity, showing a synergistic effect. The synergistically enhanced catalytic activity might be explained as follows [44,45]: Because 4-NP was π-rich in nature, it was expected that 4-NP could be adsorbed onto the surface of rGO via π-π stacking interaction, providing a higher 4-NP concentration near the Ag nanoparticles on the surface of rGO and therefore leading to the more efficient contact between them. For the earlier studies, the catalysts and/or the catalyst supports were usually present in the form of particles [1,5-10]. Also, the adsorption of 4-NP on the surface of catalyst support was not significant like that on the surface of rGO. So, the reaction occurred mainly via the collision of 4-NP molecules between catalyst particles, leading to a slower reaction rate [46].

**Figure 8:**
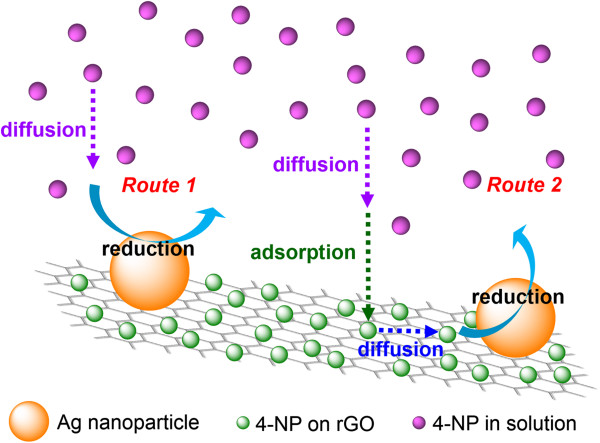
**Catalytic reduction of 4-NP with NaBH**_**4 **_**by Ag/rGO nanocomposite via both the liquid-phase and solid-phase routes.**

It was noted and interesting that the relationships of ln (*C*_*t*_/*C*_0_) vs. time remained linear during the whole reaction process examined for the effects of temperature, catalyst amount, and 4-NP concentration as shown in Figure 7a, b, d. They revealed that the *k* value remained constantly unchanged during the reaction process. This phenomenon seemed to conflict with the above effect of 4-NP concentration on the *k* value because 4-NP concentration would decrease during the reaction process and lead to the decrease of the *k* value. This could be explained reasonably by the fact that 4-NP might be reduced to 4-AP via two routes: solid phase and liquid phase. As stated above, 4-NP not only was present in the bulk solution but also might be adsorbed on the surface of rGO. During the reaction process, both the concentrations of 4-NP in the bulk solution and on the surface of rGO might decrease simultaneously. The lower 4-NP concentration on the surface of rGO would lead to the decrease of the *k* value. However, 4-NP also might diffuse to the surface of Ag nanoparticles directly from the bulk solution. Because smaller metal nanoparticles could accelerate the electron transfer owing to their higher redox potential, the heterogeneous charge transfer might be faster than diffusion [1]. So, the observed rate constant *k*_obs_ can be expressed by the Smoluchowski expression [1]:

(1) $$k_{\mathrm{obs}}\;=\;4\mathrm{\pi DR}\text{,}$$

where *D* is the diffusion coefficient and *R* is the radius of metal nanoparticles. Because the diffusion coefficient is inversely proportional to the 4-NP concentration, the *k*_obs_ value is expected to increase with the decrease of 4-NP concentration. This phenomenon was similar to that observed in some earlier studies using the catalysts and/or catalyst supports in the form of particles and could be attributed to the diffusion-controlled mechanism [1,5-10]. Thus, the opposite effects of 4-NP concentration in the solid-phase and liquid-phase routes might be counterbalanced, leading to the fact that the *k* values had no significant changes during the reaction process. Accordingly, a scheme for the catalytic reduction of 4-NP with NaBH_4_ by Ag/rGO nanocomposite via both the liquid-phase and solid-phase routes could be described by Figure 8.

All the above observations demonstrated that the resulting Ag/rGO nanocomposite exhibited good catalytic activity toward the catalytic reduction of 4-NP to 4-AP with NaBH_4_, and the support rGO also enhanced the catalytic activity via a synergistic effect. As compared to previous works, the catalytic ability of the Ag/rGO nanocomposite obtained in this work was superior or comparable to most of them although different materials and conditions were used [1,5-7,9,10,20,24-27,35,44]. This revealed that the Ag/rGO nanocomposite developed in this work could be used as a highly effective catalyst for the reduction of 4-NP with NaBH_4_.

**Figure 9:**
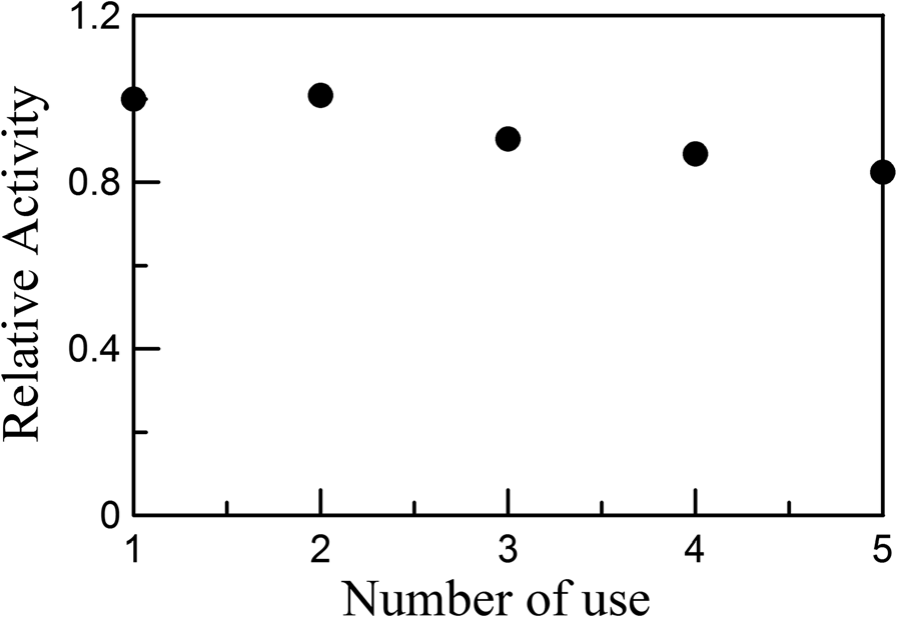
**Reusability of Ag/rGO nanocomposite for the catalytic reduction of 4-NP with NaBH**_**4**_**.** [4-NP] = 0.05 mM, [NaBH_4_]/[4-NP] = 100, catalyst amount = 0.25 mg/100 mL, temperature = 25°C.

Figure 9 shows the reusability of the Ag/rGO nanocomposite (0.25 mg/100 mL) for the catalytic reduction of 4-NP (0.05 mM) with NaBH_4_ at 25°C. It was found that 83 % of catalytic activity was retained after reuse for five times, revealing the good stability. This revealed that the Ag/rGO nanocomposite was not deactivated or poisoned significantly. The decrease of catalytic activity might be due to the loss of the Ag/rGO nanocomposite during the washing/centrifugation process because quite low catalyst concentration was used.

## Conclusions

Ag/rGO nanocomposite has been fabricated successfully via a rapid and facile green process. By the use of l-arginine and microwave irradiation, Ag nanoparticles were deposited uniformly on the surface of rGO. The Ag/rGO nanocomposite showed excellent catalytic activity and stability for the reduction of 4-NP to 4-AP with NaBH_4_. Also, the catalytic activity depended on both the size and content of Ag nanoparticles on rGO, and their appropriate controls were required. In addition, a mechanism for the catalytic reduction of 4-NP with NaBH_4_ by Ag/rGO nanocomposite via both the liquid-phase and solid-phase routes was suggested to describe the synergistic effect of rGO. Such a product could be used in the industrial wastewater treatment and the conversion of 4-NP to 4-AP in aqueous solution under mild condition.

## Competing interests

The authors declare that they have no competing interests.

## Authors’ contributions

KCH carried out the experiments and drafted the manuscript. DHC guided the study and modified the manuscript. Both authors read and approved the final manuscript.

## Authors’ information

KCH is currently a PhD student of the National Cheng Kung University (Taiwan). DHC is a distinguished professor of Chemical Engineering Department at National Cheng Kung University (Taiwan).
